# A Practical Tool for Family Assessment Based on the Social Relations Model

**DOI:** 10.3389/fpsyg.2021.699831

**Published:** 2021-07-07

**Authors:** Tom Loeys, Marieke Fonteyn, Justine Loncke

**Affiliations:** Department of Data Analysis, Ghent University, Ghent, Belgium

**Keywords:** family assessment, family social relations model, attachment security, factor scores, family systems theory

## Abstract

An empirically based family assessment can help family therapists understand how a family functions. In systemic therapy a family is seen as a dynamic system in which the family members form interdependent subsystems. The Social Relations Model (SRM) is a useful tool to study such interdependence within a family. According to the SRM, each dyadic score is viewed as the sum of an unobserved family effect, an individual actor and partner effect, and a relation-specific effect. If dyadic data are obtained for a specific family using a round robin design, these different SRM effects can be calculated using an ANOVA-approach. To gain insight into the functioning of a particular family, the family-specific SRM effects can be compared to those from a norm sample and it can be deduced whether that family has deviating scores on a particular SRM effect. Currently, such a family assessment relies on the mean and variance of the SRM ANOVA scores in the norm sample. However, family therapists may not always have access to these data, making the current approach of SRM family assessment not as useful in practice. In this article, we introduce a user-friendly web application that uses an alternative method for SRM family assessment. This alternative strategy requires as input the population parameter estimates of SRM means and variances more commonly described in SRM family literature.

## Introduction

Suppose a systemic family therapist asks the mother, the father and two children in a four-person family about their attachment security to each other. If the youngest child then reports a high score of relationship anxiety in his/her relation to his/her father, the therapist wants to be able to explain this high score. The score can be attributed to the child's personality and his/her high sense of being anxiously attached to all his/her family members. It may also reflect the father's personality and the degree to which all his family members feel anxiously attached to him. However, another possibility is that it may depend on the child's unique sense of fearful attachment to his/her father and thus not reflect the child's or father's general characteristics. Finally, it can also be explained by the family culture. It is possible that the culture of this particular family is characterized by anxious family relationships. In other words, explanations can be found on multiple levels of the family system (i.e., the personality of each family member, the relationships between the various family members, and the family as a whole).

One model that can help the family therapist to gain insight into these dynamics of a family is the Social Relations Model (SRM; Kenny and La Voie, 1984). The SRM can take into account the different levels of the family system (i.e., individual, dyadic, and family) and their complex interplay (Cook, 2000). Indeed, the SRM decomposes directed-relationships data or dyadic measurements into a family effect (at the group level), an actor and partner effect (both at the individual level) and a relation-specific effect (at the dyadic level). As explained above, the obtained rating may be determined by several factors when a child is asked to rate how he or she feels anxious attached to his or her father. First, it may be determined by the child's general perception of feeling anxiously attached to all family members. In the SRM, this factor is called the child's actor effect and reflects a cross-relational consistency in the child's feelings. The child–father measurement of relationship anxiety can be further determined by how all the family members tend to feel anxiously attached to the father. This factor is referred to as the father's partner effect and reflects a cross-relational consistency in how the other family members view the father. For example, a judgmental father may induce interpersonal anxiety from all family members, regardless of their own general dispositions to feel that way. The child–father measurement of relationship anxiety can also be determined by the child's feelings of anxious attachment to the father that is unique to their relationship. This factor is therefore called the relationship effect. Relationship effects are always directional because the relationship from the child to the father is not identical to the relationship from the father to the child (Kenny et al., 2006). Finally, it can be determined by the overall level of anxious attachment in the family. This effect is called the family effect and captures similarities among the different family members (De Mol et al., 2010).

A systematic investigation of these levels is only possible if all family members are asked the same questions according to a round robin design. In a round robin design, each family member is asked to rate a psychological concept (such as relationship anxiety) in relation to each of the other family members. These dyadic measurements act as snapshots of the family from each point of view and allow for a holistic view of the family (Cook, 2005). By using dyadic round robin data from a multidirectional perspective, we can go beyond traditional family research where the unit of analysis is limited to solely a specific family member, a specific dyad or the family as a whole (Bray, 1995; Card and Barnett, 2015).

At least four family members are needed to get a complete picture of the family. The SRM can in principle be used in families with at least three members. However, unlike families of four people, not all SRM effects described above are identified in three-person families. In addition, in this article we will focus on four-person families as most of the SRM family research considers families with four participating members (Eichelsheim et al., 2009).

The SRM is mostly used as a statistical tool by family researchers in academia to elucidate the relative importance of SRM effects as sources of variation in the dyadic measurements. This is to answer questions such as, “Do families mainly differ from each other on a specific construct, because of different actor effects (i.e., individual traits) or because of different family culture?” In other words, based on a representative sample of families, we want to make statements about family functioning in the corresponding population, using estimators for the relevant population SRM parameters. In contrast, in a therapeutic setting, the SRM effects for a particular family can help a family therapist understand that particular family functioning. For such an assessment, we must first calculate the SRM effects themselves for that particular family. Cook and Kenny (2004) have proposed ANOVA-based formulas to calculate these SRM effects for individual families. Extreme scores then indicate at what level a family may be challenged in specific family functioning. These extreme scores are discovered by evaluating how the family deviates from a norm population. This evaluation takes place in a similar way as in other “norm-referenced” psychological tests, such as, for example, an intelligence test. In this type of tests, the values of the test-takers are compared to the values of a norm sample to determine whether their individual scores are much better or worse than those of the norm sample. More specifically, a *Z* score is usually calculated by subtracting the mean of the norm sample from the relevant score and dividing by the standard deviation of the norm sample. Extreme *Z* scores (e.g., larger than two in absolute value) are indicative for outlying observations and warrant further exploration. For reliable comparisons, normative samples are often chosen based on demographical characteristics such as nationality, age or ethnicity. Moreover, a valid interpretive comparison is only possible if the norm sample has been subjected to exactly the same questionnaire.

SRM family assessment works in a similar way, as the SRM effects of a particular family are also compared to those of a norm sample. This produces *Z* scores that indicate how much the family differs from the sample (Cook, 2015) on an individual, relationship or family level. Extreme *Z* scores clearly indicate whether a family is challenged because of problems of one or more individuals (actors or partners), one or more relationships, the family as a whole, or a combination of these components (Cook and Kenny, 2004). For instance, suppose the youngest child in the specific family being assessed has a high actor effect compared to the youngest children in the families of the norm sample. In our previous example, this means that this child feels more anxiety in his or her relationships with the other family members than the average child. This observation provides the family therapist with additional information about the functioning of that child and his or her position within the family. In this sense, the SRM family assessment can function as an additional tool to the family therapist to define the level at which one would want to intervene (De Mol et al., 2010). Using this approach, Cook and Kenny (2004) thus clearly bridged the gap between SRM family research and family assessment as a clinical tool (De Bruyn, 2005).

Both procedurally and conceptually, this method differs from most other self-report family assessments. Indeed, the SRM approach to measuring the family system contrasts with more traditional procedures where one or more family members assess the family as a whole. For a long time, the most common way of assessing the family system has been to use measures whereby an individual rates the family as a whole. For example, the Family Environment Scale (FES; Moos and Moos, 1981) and the Family Adaptability and Cohesion Scales (FACES III; Olson et al., 1985) are well-known versions of whole-family assessments. As Cook and Kenny (2006) argued, such scales are problematic for a number of reasons. First, each family member can have a different perspective on how the family functions. Second, a family member can report that the family as a whole functions well despite problems in one subsystem in the family. Third, the patterns between the relationships, not the group average, define the proverbial “whole” that is greater than the sum of its parts. Finally, family dynamics often cause family members to be different as they offset each other's behavior. Existing measures of whole-family functioning do not measure or reveal such patterns.

The SRM, on the other hand, provides a map of the family system that is unrivaled in its ability to pinpoint both troublesome and beneficial aspects of family functioning. Before the development of the SRM, there was no model for family systems that simultaneously measured functioning of the individual members, their relationships with each other, and the family as a group. Although researchers collected family relationships data, there was no useful map of how the relationships fit together. The application of the SRM to round-robin family data was equivalent to the development of the microscope. For the first time, a therapist could peer into the family system, observe the elements in the system and how they relate to each other, and come to some understanding of how family roles, individual differences, interpersonal relationships, and group-level effects contribute to the quality of family life. For example, as illustrated by Cook (2005), families can be judged on a wide variety of dimensions: positivity and negativity within the domain of affectivity, effectance, and acquiescence from the domain of interpersonal control, and relationship anxiety and comfort depending on others from the domain of attachment security.

This article is not intended to discuss in detail the practical relevance of the SRM family assessment, as it has already been illustrated elsewhere (see for e.g., Cook, 2005; De Mol et al., 2010). Nor is the paper intended to describe a complete family assessment of a specific case with in-depth interpretations and possible avenues for therapeutic interventions. Rather, we strive to make the practical implementation of the SRM family assessment, which is just one of the multiple perspectives for better understanding family functioning, more accessible to therapeutic practice through a user-friendly web application that automatically performs such SRM family assessment. The web application has two advantages for therapists and researchers. First, the application only requires a minimal input from the user: the 12 dyadic measures (i.e., for each of the 12 relationships in a four-person family) for the construct of interest and the estimated population SRM parameters for that construct in the relevant population, which are usually described in the SRM family literature. This makes the application more useful for family practitioners than the current approach to SRM family assessment. Indeed, as we will explain further in the paper, the current approach requires information of the comparative sample that is often not readily available in the literature. Second, the web application performs all the calculations automatically, and thus helps the family therapists with a tedious task they otherwise would have to perform themselves.

This paper is further organized as follows. We will begin by describing an illustrative case study on relationship anxiety in a single family and will compare the dynamics of that family with a normative sample described in the literature. This case study will serve as a running example throughout this article and is also used as a textbook example in the chapter on Social Relations Designs with Roles in the book of Kenny et al. (2006). Next, we will describe how the SRM can be used in family assessment and show how to estimate the SRM effects of the individual. The section thereafter examines the population parameter estimates of the SRM means and variances that are available in the family literature, and how these can used to obtain *Z* scores. Then, based on the case study, a user-friendly free web application is presented. We end with a brief review of our findings.

## Case Study

### Description of the Individual Family

To illustrate the procedure of SRM family assessment and the accompanying web application, we look at the textbook example from Kenny et al. (2006) on the security of attachment in family relationships in a specific family. The particular family described in the book and used for illustrative purposes was randomly chosen from 208 US families who reported on their security of attachment to each other. Each member of the specific family (i.e., the mother, the father, a college student and a sibling) filled in a questionnaire with five items about relationship anxiety (RS-anxiety; Cook, 2000) toward each other member of the family. Relationship-specificity was achieved by leaving a blank in the text of the item at the point where the person's name or role would normally be indicated. Subjects were instructed to mentally insert the name of a specified family member in the blank line. The five items measuring RS-Anxiety were as follows: 1 = I feel that ___is reluctant to get as close to me as I would like; 2 = Often I worry that ___does not really love me; 3 = I want to be close to ___, but I worry that he/she will hurt me; 4 = Often I worry that ___ would like to avoid me; and 5 = I often wonder whether ___ really cares about me. Items were answered on 5-point Likert scales anchored at the extremes by 1 (strongly disagree) and 5 (strongly agree). For each of the 12 directed relationships within the family of size four, the average over the five items was then calculated. Table 1 shows these 12 dyadic measurements for the family of interest. The highest score is observed in the relationship anxiety of the youngest child toward the father. One of the questions that then may arise is for example how this high score can be explained. Is this really something specific to this relationship, or do individual or family characteristics play a role? And to what extent does that differ from what we usually see in other families?

**Table 1:** Raw dyadic measurements of the family of interest (M, mother; F, father; C1, oldest child; C2, youngest child).

**Dyadic measurement**	**Score**
M–F	1.00
M–C1	1.00
M–C2	2.00
F–M	1.17
F–C1	1.50
F–C2	3.83
C1–M	1.09
C1–F	1.33
C1–C2	2.83
C2–M	1.17
C2–F	4.83
C2–C1	3.33

### Description of the Normative Sample

In order to draw conclusions for the specific family, a comparison with a normative sample is therefore necessary. The normative sample that will be considered here is described in full detail elsewhere (Cook, 2000). In total, 208 four-person families (mother, father, a college student and a younger sibling between 12 and 19 years old) were collected through snowball sampling. The mean age was 45 years for the mothers and 49 years for the fathers. The sample of the older children consisted of 81 young men and 127 young women (mean age 19), while there were 102 brothers and 106 sisters in the sample of younger children (mean age 16). The families were mostly middle-class and Caucasian. Table 2 presents mean and standard deviation of the dyadic measurements for RS-anxiety in the normative sample (Cook, 2000). Note that the individual family has similar demographical characteristics as the normative sample. It could be tempting now to directly compare the dyadic scores from the specific family with the corresponding means in this norm population. However, as pointed out by Cook (2005), it is more interesting to explore the etiology of those dyadic scores and to use the Social Relations Model to disentangle the dyadic score into more meaningful components. In the next section we discuss the SRM decomposition in more detail.

**Table 2:** Mean and standard deviation (SD) of each dyadic measurement in the normative sample.

**Dyadic measurement**	**Mean**	**SD**
M–F	1.83	0.88
M–C1	1.75	0.71
M–C2	1.85	0.78
F–M	1.89	0.91
F–C1	1.90	0.70
F–C2	2.00	0.74
C1–M	1.48	0.62
C1–F	1.74	0.74
C1–C2	1.88	0.75
C2–M	1.73	0.73
C2–F	1.96	0.83
C2–C1	2.07	0.77

## SRM Population Parameters

**Figure 1:**
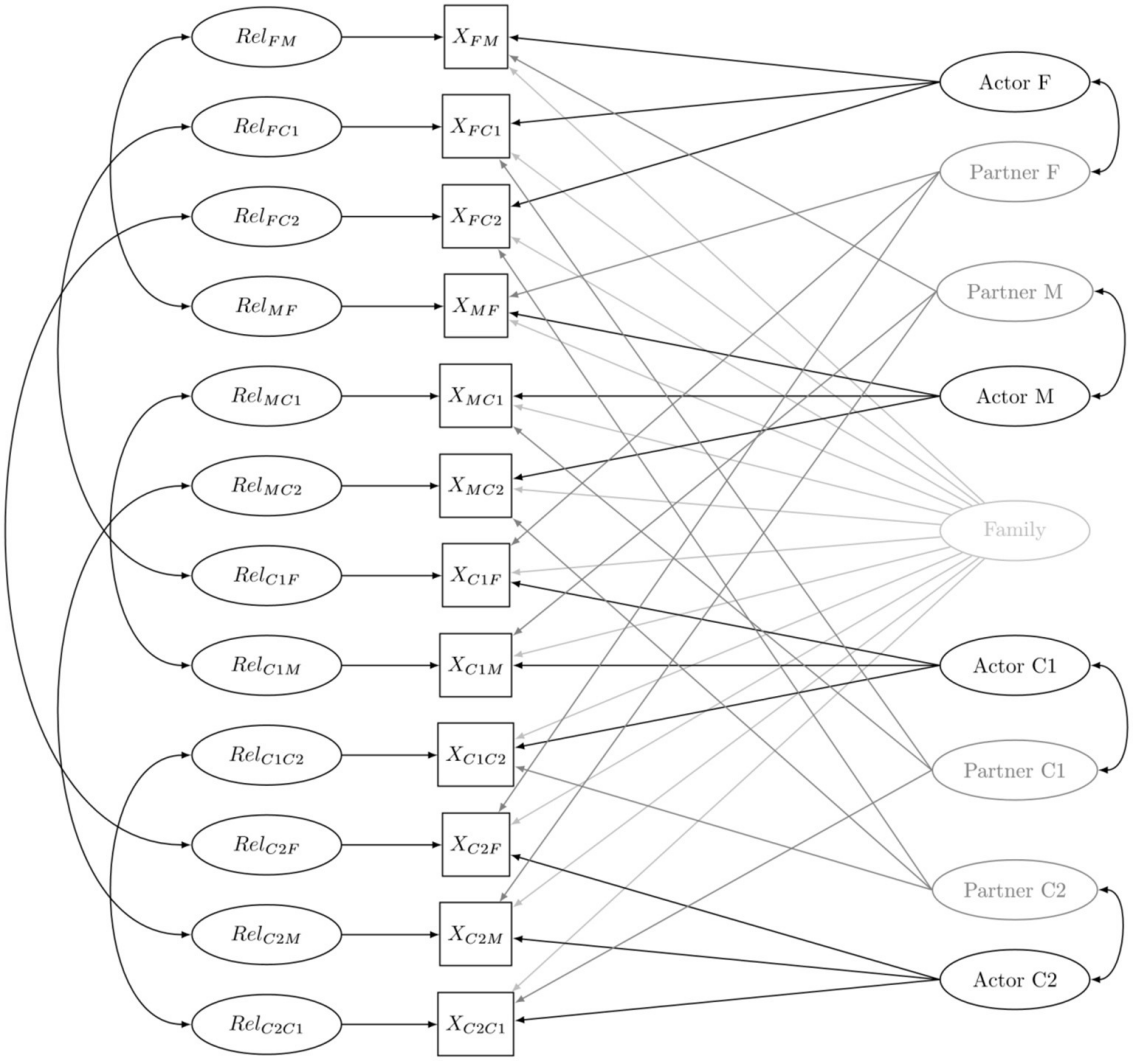
The family social relations model (M, mother; F, father; C1, oldest child; C2, youngest child).

Consider the dyadic measurement *X*_*ij*_. In a four-person family the indices *i* and *j* represent the father (F), mother (M), oldest child (C1), or youngest child (C2). *X*_*MC*__1_, for example, represent the RS-anxiety that the mother (M) experiences in relation to the oldest child (C1). According to the SRM, dyadic measures can be decomposed into four different effects at three different levels: the individual, the dyadic and the family level. More specifically, the SRM assumes that each dyadic measurement can be expressed as a linear function of an unobserved family effect *Fam*, an unobserved actor effect *Act*, an unobserved partner effect *Par* and an unobserved relation-specific effect *Rel*. Figure 1 shows the model with the SRM effects specified as latent (i.e., unobserved) variables and arrows pointing from those latent variables toward the dyadic measurements. Thus, *X*_*MC*__1_ for example can be decomposed as follows (i.e., the sum of all latent constructs that point to that dyadic score):

(1) $$\begin{array}{l} X_{MC1}=Fam+Act_{M}+Par_{C1}+Rel_{MC1} \end{array}$$

Note that there is no measurement error present in this equation. The measurement error cannot be disentangled from the relationship effect when there is only one dyadic measurement for each relationship. To separate the relationship effect from the measurement error at least two observations of each dyadic relation are needed (Back and Kenny, 2010). We will consider only one observation per dyadic measurement, and thus absorb the error in the relationship effect, since this approach is mostly used in SRM family research. Given that the relationship effects are contaminated with residual error we need to be careful in interpreting those effects (Kenny et al., 2006).

We now aim to estimate those unobserved or latent SRM components. This is usually achieved using a confirmatory factor analysis (CFA), a statistical technique that is often used for latent variable modeling. Alternatively, a multilevel approach may be used instead (Jenkins et al., 2012; Nestler, 2016). While the latter approach can more easily deal with varying family sizes (Browne et al., 2016), the former has been more commonly used by family researchers.

Interest primarily lies in the variance of the SRM components (Browne et al., 2019). Specifically, the SRM variances are used to provide answers to questions such as, “Do mothers vary across families in their anxious family relationship?” Finding a significant actor variance for mothers means that the individual characteristics of the mothers explain why they perceive different amounts of anxious attachment in their family relationships. The absence of a significant variance, on the other hand, means that the specific SRM effect is constant across families, implying that it is not a reason for variability in the observed dyadic measurements. As another example, a non-significant family variance would mean that the differences in RS-anxiety between the families are not a function of the family culture.

The SRM effects are further assumed to be independent, except for some that are related through patterns of reciprocity (Kenny et al., 2006). Generalized reciprocity reflects the correlation between a person's actor and partner effect (e.g., capturing whether the amount of anxious attachment in family relationships experienced by the mother is associated with the amount of anxious attachment that the other family members experience in relation to the mother). Dyadic reciprocity reflects the correlation between the relationship effects that represent two sides of a certain relationship (e.g., capturing whether the father–child RS-anxiety is associated with the child–father RS-anxiety). These two types of reciprocity are indicated in Figure 1 by two-headed arrows.

Additionally, the means of the SRM effects can be estimated. In a four-person SRM model with one indicator per relationship, only 12 means of the 12 dyadic measurements are observed, while there are 21 SRM means (1 family mean, 4 actor means, 4 partner means, and 12 relationship means) that need to be estimated. Consequently, constraints are needed on the mean structure to identify the model. Restrictions are typically applied such that the mean actor effects sum to zero, the mean partner effects sum to zero, and the mean SRM relationship effects sum to zero for a given actor or a given partner. Both actor and partner means then represent deviations from the family mean, which is defined as the average over the 12 dyadic measurements (Kenny et al., 2006; Eichelsheim et al., 2009). A positive mean for the actor effect of the youngest child implies that over all families the youngest child has a higher feeling of anxious attachment in relation to all other family members as compared to the average of that feeling in the family.

For the normative sample in our illustration, a CFA for the SRM was conducted to determine the main sources (i.e., family effect, actor, partner and relationship effects) of the family dynamics of attachment security (Cook, 2000). The estimated SRM means and covariances are reported in **Tables 4**, **5** [note that for consistency we reported the values from the Kenny et al. (2006) book, Dyadic Data Analysis]. We interpret a few of those effects here. The mean RS-anxiety in families equals 1.838, and so we observe on average relatively little relationship anxiety in families. There is significant but small variation (0.039) in the family effect between families. Considering the rule of thumb that ~95% of the observations lie within 2 standard deviations of the mean (assuming normality), we have that the majority of families report an average RS-anxiety in their family between 1.443 and 2.233. The mean actor effect of the youngest child equals 0.117, meaning that those children report more RS-anxiety toward all family members than the other family members. There is also substantial significant variation (0.232, *p* < 0.001) between families in the RS-anxiety toward all family members reported by those children. Those interpretations already illustrate that such information might be useful for therapists and researchers to decide at which level to intervene. Since we find significant SRM variances for all effects, a therapist or researcher might consider intervening at all levels. If a specific effect would not show significant variation, it would make no sense according to the SRM-theory to intervene at that particular level. However, to see at which level it can be useful to intervene for a specific family, we first need to know the dynamics for that specific family.

## Family Assessment Using SRM Scores

We want to explore which SRM effects form a risk factor in the family of interest. To this end we need a value for each of the unobserved SRM effects. In a two-parent two-child family, there will be 21 SRM effects: four actor effects (one for each family member), four partner effects (also one for each family member), twelve relationship-specific effects (one for each directed relationship), and lastly one family effect. Estimates for the values of each SRM effect in a specific family can easily be obtained using the 12 dyadic scores observed in that family (Cook and Kenny, 2004). More specifically, the observed dyadic values, which are presented in Table 1, are organized in a table in which the rows are the actors and the columns the partners (see Table 3). This table can be seen as a two-way analysis of variance (ANOVA) design and, therefore, the scores are often referred to as ANOVA scores. The row means represent the person's average rating of the other family members, while the column means represent the average rating of the person by the other family members. The grand mean or family mean is the average of the 12 dyadic measurements. In our specific family the family mean has a value of 2.090. Ideally, the row and column means could be considered as estimates for the actor and partner effects, respectively. However, there are empty cells present in the table, because the round robin design that we consider does not include self-ratings (Cook and Dreyer, 1984; Kenny et al., 2006). The row and column means would, therefore, reflect biased estimates of the actor and partner effects. Consequently, the means are weighted in a certain manner to take into account the missing cell. Concretely, the ANOVA scores of the actor effect and partner effect for role *i* [where *i* denotes mother {M} father {F} oldest child {C1} or youngest child {C2}] are estimated by weighing the respective row and column mean, and the grand mean, using the number of persons in the family (*n* is equal to four in the setting that we consider) i.e.,

(2) $$\begin{array}{l} \text{Ac}{\text{t}}_{\text{i}}=\frac{{\left(\text{n}-1\right)}^{2}}{\left[\text{n}\left(\text{n}-2\right)\right]}\text{row mea}{\text{n}}_{\text{i}}+\frac{\text{n}-1}{\left[\text{n}\left(\text{n}-2\right)\right]}\text{column mea}{\text{n}}_{\text{i}}\\ -\frac{\text{n}-1}{\text{n}-2}\text{ grand mean} \end{array}$$

(3) $$\begin{array}{l} \text{Pa}{\text{r}}_{\text{i}}=\frac{{\left(\text{n}-1\right)}^{2}}{\left[\text{n}\left(\text{n}-2\right)\right]}\text{column mea}{\text{n}}_{\text{i}}+\frac{\text{n}-1}{\left[\text{n}\left(\text{n}-2\right)\right]}\text{row mea}{\text{n}}_{\text{i}}\\ -\frac{\text{n}-1}{\text{n}-2}\text{ grand mean} \end{array}$$

For instance, in our specific family the actor effect of the youngest child and the partner effect of the father can be calculated as follows:

(4) $$\begin{array}{l} \text{Ac}{\text{t}}_{\text{C}2}=\frac{{\left(4-1\right)}^{2}}{\left[4\left(4-2\right)\right]}3.11+\frac{4-1}{\left[4\left(4-2\right)\right]}2.89\\ -\frac{4-1}{4-2}2.09=1.45 \end{array}$$

(5) $$\begin{array}{l} \text{Pa}{\text{r}}_{\text{F}}=\frac{{\left(4-1\right)}^{2}}{\left[4\left(4-2\right)\right]}2.39+\frac{4-1}{\left[4\left(4-2\right)\right]}2.17\\ -\frac{4-1}{4-2}2.09=0.36 \end{array}$$

Lastly, the effect for a particular relationship is obtained by subtracting the family mean, the actor and partner effect from the raw dyadic score. For instance, the relationship effect between the youngest child and the father can be calculated as follows

(6) $$\begin{array}{l} Rel_{C2F}=X_{C2F}-Act_{C2}-Par_{F}-Fam \end{array}$$

(7) $$\begin{array}{l} Rel_{C2F}=4.83-1.45-0.36-2.09=0.93 \end{array}$$

To make the scores clinically useful, they must be compared to the scores from a normative sample (Cook and Kenny, 2004). Standardized scores or *Z* scores are calculated for each of the SRM effects of that family. The *Z* score of an SRM effect in a specific family can simply be obtained by subtracting the sample mean of the SRM ANOVA scores for that effect in the norm group and dividing it by the sample's standard deviation of that SRM effect in the norm group. For example, the *Z* score for the actor effect of the mother (*Z*_*act*_*M*__) is then obtained by

(8) $$\begin{array}{l} Z_{act_{M}}=\frac{{\hat{act}}_{M_{ind}}-\;mea{n_{{\hat{act}}_{M_{norm}}}}_{\;}}{sd_{{\hat{act}}_{M_{norm}}}} \end{array}$$

where ${\hat{act}}_{M_{ind}}$ represents the estimated ANOVA score for the actor effect of the mother for the specific family, $mea{n_{{\hat{act}}_{M_{norm}}}}_{\;}$represents the norm sample's mean of the mothers' ANOVA scores for the actor effect, and $sd_{{\hat{act}}_{M_{norm}}}$ represents the norm sample's standard deviation of the mothers' ANOVA scores for the actor effect.

**Table 3:** Raw dyadic scores according to a two-way ANOVA design (M, mother; F, father; C1, oldest child; C2, youngest child).

**Actor**	**Partner**				
	**M**	**F**	**C1**	**C2**	**Row mean**
M		1.00	1.00	2.00	1.33
F	1.17		1.50	3.83	2.17
C1	1.09	1.33		2.83	1.75
C2	1.17	4.83	3.33		3.11
Column mean	1.14	2.39	1.94	2.89	2.09

By standardizing the SRM effects, the *Z* scores all have a mean of zero and a standard deviation of one. Such a *Z* score deviates significantly from the norm mean when it is more than two standard deviations above or below that mean (Cook and Kenny, 2004). Meaning that a *Z* score of ±2 for a particular SRM effect indicates an extreme deviation (De Mol et al., 2010). For example, when the actor effect of the mother has a *Z* score of more than 2, this means that the mother reports more anxiety in her family relationships compared to the mothers from the normative sample. Or the opposite is also possible, for example, when the partner effect of the father has a *Z* score of <-2, this means that the father is experienced by his family members as eliciting less anxiety compared to fathers from the normative sample. Note that a *Z* score of more than one standard deviation above or below the mean (i.e., a *Z* score of ±1) may also indicate a risk factor. These SRM effects should therefore still be taken under further consideration by the clinical practitioner. *Z* scores may, however, only be interpreted for SRM effects that have a significant and relevant variance in the normative sample (Cook and Kenny, 2004). Note that the SRM effects can be extreme even when the raw dyadic measures are not, showing that they provide both greater sensitivity and specificity than the raw dyadic measurements of the family relationships (Cook, 2005).

The above described procedure to calculate the *Z* scores makes use of the means and standard deviations of the norm sample's ANOVA scores. The latter can only be calculated when one has access to the raw dyadic measurements of the normative sample or when the SRM ANOVA means and standard deviations in the norm sample are presented in a paper. However, the latter are typically not published in family literature. Additionally, an individual family therapist often has no access to the raw dyadic measurements of the normative sample. Typically, we only have access to the information on the normative sample such as described in Cook (2000). That is, the means and variances of the SRM effects based on a CFA are reported, but those do not necessarily equal the means and variances of the ANOVA SRM effects.

Thus, how can we calculate, for example, the *Z* score of the mother's actor effect when we do not know $mea{n_{{\hat{act}}_{M_{norm}}}}_{\;}$ and $sd_{{\hat{act}}_{M_{norm}}}$? Would it be possible to calculate the *Z* scores of a particular family by using the CFA population parameter estimates for the SRM means and variances that are more frequently described in the family literature?

The answer to this question is yes. The link between the estimators of the means and variances of latent effects using CFA and the estimators of the mean and variances using ANOVA is known in a general setting (Hoshino and Bentler, 2011). A detailed discussion of this transformation falls outside the scope of this article, but the interested reader can find a detailed derivation on the link between both for the specific SRM setting in the Appendix. By transforming the SRM means and SRM variances that are obtained from the CFA and that are typically reported in the literature to the means and variances of the SRM ANOVA effects, we will be able to obtain correct *Z* scores for the individual ANOVA effects for a specific family. We skip the technical details here (see Appendix), but rather focus in the next section on its practical implementation in a user-friendly free web application.

## Family Assessment Using the Application

In this section we evaluate for our case study which SRM effects in the specific family deviate from the norm sample. We do not go into much detail about the clinical case here and refer the family therapist interested in a deeper understanding of SRM assessment as a clinical tool to comprehensive case studies described elsewhere (Cook, 2005; De Mol et al., 2010). The case study discussed in Cook (2005) consists of a family with a mother and father who are separated for 8 years, a daughter who is 18, and the son (age 16) who is the patient (major depression). Three domains of family functioning were assessed, each containing two dimensions (Positive and Negative Affectivity, Interpersonal Effectance and Acquiescence, Relationship-Specific Attachment Security). De Mol et al. (2010) describes a family consisting of a 40-year-old mother, a 55-year-old stepfather, a daughter aged 16, and a daughter (aged 15) who has been hospitalized for the past year in a child psychiatric center. The subject of the family assessment was family members' sense of influence in their family relationships. The younger daughter was hospitalized because of severe aggressive behavior toward all other family members and outside the home.

In this paper, we rather focus on the practical use of the app. More specifically, we explain how one can obtain *Z* scores for assessment based on the raw dyadic measurements of the family of interest and the population parameter CFA estimates of the SRM means and variances in the normative sample. An online application was built within the statistical software *R* using package shiny (Chang et al., 2017; R Development Core Team, 2017). This user-friendly and free application can be found at https://srmfamilyassessment.shinyapps.io/Zscores/. Note that although *R* is used in the background of the application, the user does not need to install *R* on his or her computer. Before presenting the results of the SRM family assessment, we will first describe the different steps of the application.

In a first step and consequently in the first tab, the user is asked to choose labels for the different roles of the family members. In this case one can opt for the following labels: M (mother), F (father), oldest child (C1), and youngest child (C2). The other tabs of the application are then automatically adapted according to these choices of the user. Next, one needs to enter the 12 raw dyadic values observed in the family of interest. These values are often the mean or the sum scores of a subscale's items of a questionnaire. Here, the raw scores are the mean scores of the RS-anxiety scale per dyadic relationship (see Table 1). In a third step, one needs to enter all the population parameter estimates for the SRM effects of the normative sample obtained with a CFA, namely the means, the variances and the covariances. The user is asked to first insert the estimated means and variances of the main SRM effects (i.e., the family effect, the four actor effects and the four partner effects) and then the means and variances of the twelve relationship effects. In this tab, the user is also requested to provide the generalized and dyadic reciprocities and to indicate whether the reported reciprocities are correlations or covariances. The SRM population parameter estimates of the normative sample are presented in Tables 4, 5 and were obtained using a CFA. In the last tab, the user then automatically gets the results of the SRM family assessment. That is, behind the scenes the CFA estimators for the SRM means and variances are transformed into ANOVA estimators. The user directly sees the SRM ANOVA scores for the SRM effects of the individual family, their *Z* scores as well as their accompanying *p*-values (Table 6). Note that the *Z* scores and *p*-values reported in Cook and Kenny (2006, p. 259) might slightly deviate from the values reported here due to rounding, and that in contrast to Kenny et al. (2006) we did not use the means and variances of the ANOVA-scores.

**Table 4:** Variance and mean estimates of the normative sample's SRM components (*p*-values are based on one-sided and two-sides *z*-tests for the variance and the mean, respectively, *p < 0.05; **p < 0.01; ***p < 0.001).

**SRM component**	**Variance**	**Mean**
Family	0.039*	1.838***
Actor M	0.163***	−0.087**
Actor F	0.217***	0.103**
Actor C1	0.215***	−0.134***
Actor C2	0.232***	0.117***
Partner M	0.044**	−0.169***
Partner F	0.056**	0.038
Partner C1	0.064***	0.022
Partner C2	0.079***	0.109***
Relationship M–F	0.491***	0.040
Relationship M–C1	0.223***	−0.028
Relationship M–C2	0.338***	−0.012
Relationship F–M	0.616***	0.116***
Relationship F–C1	0.170***	−0.066
Relationship F–C2	0.205***	−0.050*
Relationship C1–M	0.107***	−0.059**
Relationship C1–F	0.204***	−0.003
Relationship C1–C2	0.205***	0.062**
Relationship C2–M	0.204***	−0.057*
Relationship C2–F	0.336***	−0.037
Relationship C2–C1	0.356***	0.094***

**Table 5:** Reciprocity correlations of the normative sample's SRM components (*p*-values are based on 2-sided *z*-test: *p < 0.05; **p < 0.01; ***p < 0.001).

	**Estimate**
**Generalized reciprocity**	
M	0.40
F	0.03
C1	0.51**
C2	0.54***
**Dyadic reciprocity**	
M–F	0.35***
M–C1	0.16
M–C2	−0.00
F–C1	0.22
F–C2	0.19
C1–C2	0.05

**Table 6:** ANOVA scores, *Z* scores and *p*-values of the family of interest's family assessment (M, mother; F, father; C1, oldest child; C2, youngest child).

**SRM effect**	**ANOVA score**	***Z*-score**	***p*-value**
Family	2.090	0.639	0.523
Actor M	−1.206	−2.358	0.018
Actor F	0.198	0.191	0.849
Actor C1	−0.438	−0.628	0.530
Actor C2	1.446	2.607	0.009
Partner M	−1.349	−3.385	0.001
Partner F	0.363	0.896	0.370
Partner C1	−0.292	−0.853	0.393
Partner C2	1.279	3.151	0.002
Relationship M–F	−0.246	−0.767	0.443
Relationship M–C1	0.409	1.304	0.192
Relationship M–C2	−0.163	−0.436	0.663
Relationship F–M	0.231	0.305	0.761
Relationship F–C1	−0.497	−1.350	0.177
Relationship F–C2	0.264	0.989	0.323
Relationship C1–M	0.786	2.856	0.004
Relationship C1–F	−0.685	−2.203	0.028
Relationship C1–C2	−0.101	−0.481	0.631
Relationship C2–M	−1.018	−2.910	0.004
Relationship C2–F	0.931	2.803	0.005
Relationship C2–C1	0.086	−0.02	0.984

We now interpret those results. As already noted before, all SRM variances are significant at the 0.05 level, which implies that actor and partner effects, relationship-specific effects as well as family culture all can explain differences between families. It is hence useful to interpret all deviating effects in the specific family. Firstly, two extreme actor effects can be found. The actor effects can tell us more about the characteristics of a specific member of the family, independent from specific relationships within the family and over and above the family culture. An extreme actor effect can be found for the mother (actor effect mother *Z* = −2.358), which indicates that the mother experiences less RS anxiety than the average mother in the norm sample does. The opposite pattern is observed for the youngest child. The youngest child, a girl, experiences a lot of RS anxiety in her family relationships (actor effect youngest child *Z* = 2.607) in comparison to the youngest children in the norm sample. In addition, extreme partner effects for the mother and the youngest child are found. The deviating partner effect of the mother (*Z* = −3.385) suggests that the mother elicits less RS anxiety in her family relations compared to mother in the norm sample. On the other hand, the youngest child elicits a lot of RS anxiety from her family members (partner effect youngest child *Z* = 3.151) compared to her peers in the norm sample. The relationship effects measure how much of the perceived RS anxiety is unique to the specific relationship between two family members. In this specific family four extreme scores were found. The youngest child feels less RS anxiety in relation to her mother (relationship effect youngest child–mother *Z* = −2.91), whereas she experiences more RS anxiety in relation to her father (relationship effect youngest child–father *Z* = 2.803) in comparison to families in the norm sample. Lastly, opposite patterns are found for the oldest child as compared to the youngest child. The oldest child in this specific family feels more RS anxiety in relation to the mother than peers in the norm sample (relationship effect oldest child–mother *Z* = 2.856), whereas the oldest child feels less RS anxiety in relation to the father (relationship effect oldest child–father Z = −2.203). For this family, no other *Z* scores larger than 2 in absolute value were found. A *Z* score larger than 2 corresponds with a *p*-value smaller than 0.05. As we are looking at multiple tests (21 effects in total), it might happen that we are identifying false positives. The tool should however merely be viewed as a screening tool that can identify potential issues, and those findings should always be corroborated by other qualitative or quantitative assessments made by the family therapist.

Overall, this SRM family assessment provides some helpful insights into family functioning and complexity for a family therapist. The SRM assessment may indicate some possible avenues that may be worth exploring further to understand the family members' experiences of RS anxiety. For instance, does the youngest child's general tendency to feel high RS anxiety in relation to the other family members indicate some specific characteristics of the child in this specific family? Are there specific characteristics of the child that make the other family members experience high RS anxiety toward him/her? Or are there some characteristics of the youngest child that make him/her anxious in all his/her family relations? Or are the unique relationships within this family more important and is it the case, for example, that the youngest child feels more comfortable with the mother and feels more RS anxiety in relation to the father, while the opposite pattern is present for the oldest child?

Thus, these hypotheses based on the information obtained from the SRM assessment may further enable a therapist to gain insight into this family. The SRM assessment does indeed provide the therapist with additional information about the behavior of the different family members as well as their position in the family. Note that results of the SRM family assessment should always be interpreted with respect to the specific circumstances of the family itself. In this sense, SRM family assessment serves merely as a guide for the family therapist and is only intended to complement other psychological assessments or interview-based methodologies (De Mol et al., 2010).

## Discussion

This paper introduced a user-friendly application for SRM family. It builds on the approach to SRM family assessment originally proposed by Cook and Kenny (2004) but eradicates the need of the norm sample's mean and variance of SRM ANOVA scores. Instead, it uses the population parameter estimates of the SRM means and variances commonly described in SRM family research. However, those CFA mean and variance estimates cannot simply be used as a substitute for the ANOVA means and variances. They should be transformed to equal the mean and variance that would be obtained when using the norm group's SRM ANOVA scores. The application presented in this paper performs these calculations automatically.

To make this approach more useful in the therapeutic setting, we urge academic family researchers to share more detailed results from their SRM analyses. Because family researchers were mainly interested in the relative importance of the SRM effects as sources of variation in the dyadic measurements, they were often inclined to only report the (co-)variances (Cook, 2000) in the past. As Eichelsheim et al. (2011) also emphasized he relevance of SRM means, more recent SRM analyses now also report these means. Thanks to specific software tools that facilitate SRM analyses (Stas et al., 2015) and automatically deliver SRM means and (co)variances, we hope to see even more papers reporting both estimated means and variance from a CFA-analysis in the future.

While the case study described in this paper focused on relationship anxiety as the measurement of interest, different aspects of family functioning have been explored in the SRM family literature. Cook (2001) distinguishes two broad domains of family relations: affectivity, which includes concepts as support, attachment and family negativity, and influence which refers to concepts such as control and persuasion. Eichelsheim et al. (2009) provide a nice overview on the different family measures that have been studied using the SRM with a short description of the sample characteristics. An up-to-date bibliography of the SRM can be found at http://www.davidakenny.net/doc/srmbiblio.pdf.

To further increase the usability of the app, family researchers who have sample questionnaires available and the SRM population parameter estimates needed for assessment, and who are willing to share all that information, are invited to contact the first author. We can then make these questionnaires available through the app, as well as a description of the reference population. We can also program the SRM population parameters for those constructs into the app so that they no longer need to be entered by the family therapist who wants to use the app for their assessment. This will make the app a dynamic tool from and for family psychologists.

It is important to note that the proposed application to perform SRM family assessment does not take into account the potential impact of external variables, such as gender and age. It is known that such external variables can have an effect on the SRM effects and thus even explain a possible deviation of a certain family for a specific SRM effect (Cook, 1993). A further extension of the application could take into account the effects of such external variables. Another limitation of the current app is that it is limited to four-person families. One can of course recruit bigger families, but the SRM assessment can only take into account four members. As the vast majority of SRM-studies relies on four-person families, it made sense to build the app for this setting.

In sum, we hope that both practitioners and researchers will find the web application a useful additional tool for their family assessment. However, it is important to keep in mind that the results of the SRM family assessment should not replace other qualitative or quantitative assessments but are only intended to complement the researcher's and therapist's toolbox (De Mol et al., 2010).

## Data Availability Statement

Publicly available datasets were analyzed in this study. This data can be found at: Data are described in the book Dyadic Data Analysis by Cook and Kenny (2006).

## Author Contributions

TL suggested the idea and reviewed the paper. MF described the analysis of the case study. JL wrote the initial version of the paper and implemented the app. All authors contributed to the article and approved the submitted version.

## Conflict of Interest

The authors declare that the research was conducted in the absence of any commercial or financial relationships that could be construed as a potential conflict of interest.
