# Supplementary material for: A Practical Tool for Family Assessment Based on the Social Relations Model
Source: Front Psychol. 2021 Jul 7;12:699831. doi: 10.3389/fpsyg.2021.699831 (PMC8292793; doi:10.3389/fpsyg.2021.699831)
Supplement: Supplementary file 1 [file Data_Sheet_1.pdf]

SUPPLEMENTARY MATERIAL

1.1 SRM population parameters

According to the SRM, the dyadic measurement X_{ij}, can be decomposed into four different effects at three different levels: the individual, the dyadic and the family level. In a four-person family the indices *i* and *j* might represent the father (F), mother (M), oldest child (C1) or youngest child (C2). X_{MC1} might, for example, represent the negative influence that the mother experiences from oldest child. More specifically, the SRM assumes that each dyadic measurement can be expressed as a linear function of an unobserved or latent family effect *Fam*, a latent actor effect *Act*, a latent partner effect *Par* and a latent relation-specific effect *Rel*. Thus X_{MC1} can, for example, be decomposed as follows

$$X_{MC1} = Fam + Act_M + Par_{C1} + Rel_{MC1}$$

In CFA-notation, the raw dyadic scores X can be expressed in terms of the factor loading matrix Λ and the latent SRM effects f and the measurement error ϵ

$$X = \Lambda f + \epsilon \quad (5)$$

with

$$\mathrm{E}(f) = \nu \quad (6)$$

$$\mathrm{var}(f) = \Psi \quad (7)$$

$$\mathrm{E}(\epsilon) = 0 \quad (8)$$

$$\mathrm{var}(\epsilon) = \Theta = 0 \quad (9)$$

where ν refers to the SRM means, Ψ to the SRM covariance matrix and Θ to the residual covariance matrix. Estimates for the population parameters for the SRM variances Ψ and SRM means ν are often reported in family literature. Note that the covariance matrix Ψ contains all the SRM variances and the reciprocities, while the residual covariance matrix Θ is zero in our setting. This, because the relationship effects and measurement errors are entangled.

In the following, the exact structure of the population matrices of the SRM are defined. First, the transpose of the factor loading matrix Λ' can be written as

$$
\Lambda' = \left[\begin{array}{cccccccccccc|c}
X_{MF} & X_{MC1} & X_{MC2} & X_{FM} & X_{FC1} & X_{FC2} & X_{C1M} & X_{C1F} & X_{C1C2} & X_{C2M} & X_{C2F} & X_{C2C1} & \\
1 & 1 & 1 & 1 & 1 & 1 & 1 & 1 & 1 & 1 & 1 & 1 & Fam \\
1 & 1 & 1 & 0 & 0 & 0 & 0 & 0 & 0 & 0 & 0 & 0 & Act_M \\
0 & 0 & 0 & 1 & 1 & 1 & 0 & 0 & 0 & 0 & 0 & 0 & Act_F \\
0 & 0 & 0 & 0 & 0 & 0 & 1 & 1 & 1 & 0 & 0 & 0 & Act_{C1} \\
0 & 0 & 0 & 0 & 0 & 0 & 0 & 0 & 0 & 1 & 1 & 1 & Act_{C2} \\
0 & 0 & 0 & 1 & 0 & 0 & 1 & 0 & 0 & 1 & 0 & 0 & Par_M \\
1 & 0 & 0 & 0 & 0 & 0 & 0 & 1 & 0 & 0 & 1 & 0 & Par_F \\
0 & 1 & 0 & 0 & 1 & 0 & 0 & 0 & 0 & 0 & 0 & 1 & Par_{C1} \\
0 & 0 & 1 & 0 & 0 & 1 & 0 & 0 & 1 & 0 & 0 & 0 & Par_{C2} \\
1 & 0 & 0 & 0 & 0 & 0 & 0 & 0 & 0 & 0 & 0 & 0 & Rel_{MF} \\
0 & 1 & 0 & 0 & 0 & 0 & 0 & 0 & 0 & 0 & 0 & 0 & Rel_{MC1} \\
0 & 0 & 1 & 0 & 0 & 0 & 0 & 0 & 0 & 0 & 0 & 0 & Rel_{MC2} \\
0 & 0 & 0 & 1 & 0 & 0 & 0 & 0 & 0 & 0 & 0 & 0 & Rel_{FM} \\
0 & 0 & 0 & 0 & 1 & 0 & 0 & 0 & 0 & 0 & 0 & 0 & Rel_{FC1} \\
0 & 0 & 0 & 0 & 0 & 1 & 0 & 0 & 0 & 0 & 0 & 0 & Rel_{FC2} \\
0 & 0 & 0 & 0 & 0 & 0 & 1 & 0 & 0 & 0 & 0 & 0 & Rel_{C1M} \\
0 & 0 & 0 & 0 & 0 & 0 & 0 & 1 & 0 & 0 & 0 & 0 & Rel_{C1F} \\
0 & 0 & 0 & 0 & 0 & 0 & 0 & 0 & 1 & 0 & 0 & 0 & Rel_{C1C2} \\
0 & 0 & 0 & 0 & 0 & 0 & 0 & 0 & 0 & 1 & 0 & 0 & Rel_{C2M} \\
0 & 0 & 0 & 0 & 0 & 0 & 0 & 0 & 0 & 0 & 1 & 0 & Rel_{C2F} \\
0 & 0 & 0 & 0 & 0 & 0 & 0 & 0 & 0 & 0 & 0 & 1 & Rel_{C2C1}
\end{array}\right]
$$

Further, on the next page one can find the covariance matrix Ψ. There σ^2_{FE} measures the family variance, $\sigma^2_{A_i}$ measures the actor variance of role i, $\sigma^2_{P_i}$ measures the partner variance of role *i* (where *i* may be the father (F), mother (M), oldest child (C1) or youngest child (C2)) , σ_i measures the generalized reciprocity for role i, σ^2_{ij} measures the relationship variance between *i* and *j* and $\sigma_{i/j}$ measures the dyadic reciprocity

SUPPLEMENTARY MATERIAL

$$
\Psi = \begin{bmatrix}
\sigma^2_{FE} & 0 & 0 & 0 & 0 & 0 & 0 & 0 & 0 & 0 & 0 & 0 & 0 & 0 & 0 & 0 & 0 & 0 & 0 & 0 & 0 \\
0 & \sigma^2_{A_M} & 0 & 0 & 0 & \sigma_M & 0 & 0 & 0 & 0 & 0 & 0 & 0 & 0 & 0 & 0 & 0 & 0 & 0 & 0 & 0 \\
0 & 0 & \sigma^2_{A_F} & 0 & 0 & 0 & \sigma_F & 0 & 0 & 0 & 0 & 0 & 0 & 0 & 0 & 0 & 0 & 0 & 0 & 0 & 0 \\
0 & 0 & 0 & \sigma^2_{A_{C1}} & 0 & 0 & 0 & \sigma_{C1} & 0 & 0 & 0 & 0 & 0 & 0 & 0 & 0 & 0 & 0 & 0 & 0 & 0 \\
0 & 0 & 0 & 0 & \sigma^2_{A_{C2}} & 0 & 0 & 0 & \sigma_{C2} & 0 & 0 & 0 & 0 & 0 & 0 & 0 & 0 & 0 & 0 & 0 & 0 \\
0 & \sigma_M & 0 & 0 & 0 & \sigma^2_{P_M} & 0 & 0 & 0 & 0 & 0 & 0 & 0 & 0 & 0 & 0 & 0 & 0 & 0 & 0 & 0 \\
0 & 0 & \sigma_F & 0 & 0 & 0 & \sigma^2_{P_F} & 0 & 0 & 0 & 0 & 0 & 0 & 0 & 0 & 0 & 0 & 0 & 0 & 0 & 0 \\
0 & 0 & 0 & \sigma_{C1} & 0 & 0 & 0 & \sigma^2_{P_{C1}} & 0 & 0 & 0 & 0 & 0 & 0 & 0 & 0 & 0 & 0 & 0 & 0 & 0 \\
0 & 0 & 0 & 0 & \sigma_{C2} & 0 & 0 & 0 & \sigma^2_{P_{C2}} & 0 & 0 & 0 & 0 & 0 & 0 & 0 & 0 & 0 & 0 & 0 & 0 \\
0 & 0 & 0 & 0 & 0 & 0 & 0 & 0 & 0 & \sigma^2_{MF} & 0 & 0 & \sigma_{M/F} & 0 & 0 & 0 & 0 & 0 & 0 & 0 & 0 \\
0 & 0 & 0 & 0 & 0 & 0 & 0 & 0 & 0 & 0 & \sigma^2_{MC1} & 0 & 0 & 0 & 0 & \sigma_{M/C1} & 0 & 0 & 0 & 0 & 0 \\
0 & 0 & 0 & 0 & 0 & 0 & 0 & 0 & 0 & 0 & 0 & \sigma^2_{MC2} & 0 & 0 & 0 & 0 & 0 & 0 & \sigma_{M/C2} & 0 & 0 \\
0 & 0 & 0 & 0 & 0 & 0 & 0 & 0 & 0 & \sigma_{M/F} & 0 & 0 & \sigma^2_{FM} & 0 & 0 & 0 & 0 & 0 & 0 & 0 & 0 \\
0 & 0 & 0 & 0 & 0 & 0 & 0 & 0 & 0 & 0 & 0 & 0 & 0 & \sigma^2_{FC1} & 0 & 0 & \sigma_{F/C1} & 0 & 0 & 0 & 0 \\
0 & 0 & 0 & 0 & 0 & 0 & 0 & 0 & 0 & 0 & 0 & 0 & 0 & 0 & \sigma^2_{FC2} & 0 & 0 & 0 & 0 & \sigma_{F/C2} & 0 \\
0 & 0 & 0 & 0 & 0 & 0 & 0 & 0 & 0 & 0 & \sigma_{M/C1} & 0 & 0 & 0 & 0 & \sigma^2_{C1M} & 0 & 0 & 0 & 0 & 0 \\
0 & 0 & 0 & 0 & 0 & 0 & 0 & 0 & 0 & 0 & 0 & 0 & 0 & \sigma_{F/C1} & 0 & 0 & \sigma^2_{C1F} & 0 & 0 & 0 & 0 \\
0 & 0 & 0 & 0 & 0 & 0 & 0 & 0 & 0 & 0 & 0 & 0 & 0 & 0 & 0 & 0 & 0 & \sigma^2_{C1C2} & 0 & 0 & \sigma_{C1/C2} \\
0 & 0 & 0 & 0 & 0 & 0 & 0 & 0 & 0 & 0 & 0 & \sigma_{M/C2} & 0 & 0 & 0 & 0 & 0 & 0 & \sigma^2_{C2M} & 0 & 0 \\
0 & 0 & 0 & 0 & 0 & 0 & 0 & 0 & 0 & 0 & 0 & 0 & 0 & 0 & \sigma_{F/C2} & 0 & 0 & 0 & 0 & \sigma^2_{C2F} & 0 \\
0 & 0 & 0 & 0 & 0 & 0 & 0 & 0 & 0 & 0 & 0 & 0 & 0 & 0 & 0 & 0 & 0 & \sigma_{C1/C2} & 0 & 0 & \sigma^2_{C2C1}
\end{bmatrix}
$$

1.2 Link between SRM population parameters and ANOVA factor scores means and variances

Typically, estimation of the population parameters for the SRM variances Ψ and SRM means ν relies on structural equation modeling (SEM) by approaching the SRM-analysis as a confirmatory factor analysis (CFA; Kenny et al., 2006; Stas et al., 2015). When using maximum likelihood estimation, it renders asymptotically unbiased estimates of the SRM's true means and variances (Nestler, 2016). To evaluate whether we can use those population parameter estimates of Ψ and ν instead of the mean and variance of the SRM ANOVA scores, we will investigate the association between them.

To this end, we will first introduce an alternative expression for the SRM ANOVA scores. Namely, the ANOVA estimates of the SRM effects can also be obtained by using a weight matrix W, that is composed on basis of the aforementioned formulas of Cook & Kenny (2004). Such weight matrix for a four-person family can be defined as

X_{MF}	X_{MC1}	X_{MC2}	X_{FM}	X_{FC1}	X_{FC2}	X_{C1M}	X_{C1F}	X_{C1C2}	X_{C2M}	X_{C2F}	X_{C2C1}	
1/12	1/12	1/12	1/12	1/12	1/12	1/12	1/12	1/12	1/12	1/12	1/12	Fam
1/4	1/4	1/4	0	−1/8	−1/8	0	−1/8	−1/8	0	−1/8	−1/8	Act_M
0	−1/8	−1/8	1/4	1/4	1/4	−1/8	0	0 − 1/8	−1/8	0	−1/8	Act_F
−1/8	0	−1/8	−1/8	0	−1/8	1/4	1/4	1/4	−1/8	−1/8	0	Act_{C1}
−1/8	−1/8	0	−1/8	−1/8	0	−1/8	−1/8	0	1/4	1/4	1/4	Act_{C2}
0	0	0	1/4	−1/8	−1/8	1/4	−1/8	−1/8	1/4	−1/8	−1/8	Par_M
1/4	−1/8	−1/8	0	0	0	−1/8	1/4	−1/8	−1/8	1/4	−1/8	Par_F
−1/8	1/4	−1/8	−1/8	1/4	−1/8	0	0	0	−1/8	0 − 1/8	1/4	Par_{C1}
−1/8	−1/8	1/4	−1/8	−1/8	1/4	−1/8	−1/8	1/4	0	0	0	Par_{C2}
5/12	−5/24	−5/24	−1/12	1/24	1/24	1/24	−5/24	1/6	1/24	−5/24	1/6	Rel_{MF}
−5/24	5/12	−5/24	1/24	−5/24	1/6	−1/12	1/24	1/24	1/24	1/6	−5/24	Rel_{MC1}
−5/24	−5/24	5/12	1/24	1/6	−5/24	1/24	1/6	−5/24	−1/12	1/24	1/24	Rel_{MC2}
−1/12	1/24	1/24	5/12	−5/24	−5/24	−5/24	1/24	1/6	−5/24	1/24	1/6	Rel_{FM}
1/24	−5/24	1/6	−5/24	5/12	−5/24	1/24	−1/12	1/24	1/6	1/24	−5/24	Rel_{FC1}
1/24	1/6	−5/24	−5/24	−5/24	5/12	1/6	1/24	−5/24	1/24	−1/12	1/24	Rel_{FC2}
1/24	−1/12	1/24	−5/24	1/24	1/6	5/12	−5/24	−5/24	−5/24	1/6	1/24	Rel_{C1M}
−5/24	1/24	1/6	1/24	−1/12	1/24	−5/24	5/12	−5/24	1/6	−5/24	1/24	Rel_{C1F}
1/6	1/24	−5/24	1/6	1/24	−5/24	−5/24	−5/24	5/12	1/24	1/24	−1/12	Rel_{C1C2}
1/24	1/24	−1/12	−5/24	1/6	1/24	−5/24	1/6	1/24	5/12	−5/24	−5/24	Rel_{C2M}
−5/24	1/6	1/24	1/24	1/24	−1/12	1/6	−5/24	1/24	−5/24	5/12	−5/24	Rel_{C2F}
1/6	−5/24	1/24	1/6	−5/24	1/24	1/24	1/24	−1/12	−5/24	−5/14	5/12	Rel_{C2C1}

By multiplying this weight matrix with the vector X that contains all the raw dyadic scores, the ANOVA estimates for the SRM effects, denoted as $\hat{f}$, can be obtained as follows

$$\hat{f} = WX \tag{12}$$

Given model (5), the expectation of the estimated scores $E(\hat{f})$ can be calculated as (Hoshino & Bentler, 2011):

$$E(\hat{f}) = WE(X)$$

$$= W\Lambda E(f) \tag{13}$$

$$= W\Lambda\nu$$

where $E(X)$ refers to the expectation of the observed dyadic measurements and ν to the expectation of the latent SRM effects. By taking into account the constraints on the mean structure of the SRM, it can easily be shown that the expectation of the ANOVA scores coincide with the SRM factor means, i.e. , $E(\hat{f}) = \nu$. Because of this, the sample's means of the SRM ANOVA scores in a norm group can be replaced by the estimates of the SRM means that are obtained through a CFA in the calculation of the Z scores.

In what follows, we will present a detailed derivation for an actor and a relationship effect. This because, the derivation of the actor and partner effects are similar. For these derivations, one needs to take into account the constraints on the mean structure of the SRM. In the SRM, ANOVA constraints are typically applied such that the mean actor effects sum to zero, the mean partner effects sum to zero, and the mean SRM relationship effects sum to zero for a given actor or a given partner. This means that an actor effect can be defined in function of the other actor effects and that a relationship effect can be defined in terms of other relationship effects. Given equation (13), which embodies the relation between the means of the ANOVA scores and the SRM factor means, a first step will be to calculate the product between the weight matrix W and the factor loading matrix Λ. This product is presented on the next page.

SUPPLEMENTARY MATERIAL

$$
W\Lambda\nu = \begin{bmatrix}
1 & 1/4 & 1/4 & 1/4 & 1/4 & 1/4 & 1/4 & 1/4 & 1/4 & 1/12 & 1/12 & 1/12 & 1/12 & 1/12 & 1/12 & 1/12 & 1/12 & 1/12 & 1/12 & 1/12 & 1/12 \\
0 & 3/4 & -1/4 & -1/4 & -1/4 & 0 & 0 & 0 & 0 & 1/4 & 1/4 & 1/4 & 0 & -1/8 & -1/8 & 0 & -1/8 & -1/8 & 0 & -1/8 & -1/8 \\
0 & -1/4 & 3/4 & -1/4 & -1/40 & 0 & 0 & 0 & 0 & 0 & -1/8 & -1/8 & 1/4 & 1/4 & 1/4 & -1/8 & 0 & -1/8 & -1/8 & 0 & -1/8 \\
0 & -1/4 & -1/4 & 3/4 & -1/4 & 0 & 0 & 0 & 0 & -1/8 & 0 & -1/8 & -1/8 & 0 & -1/8 & 1/4 & 1/4 & 1/4 & -1/8 & -1/8 & 0 \\
0 & -1/4 & -1/4 & -1/4 & 3/4 & 0 & 0 & 0 & 0 & -1/8 & -1/8 & 0 & -1/8 & -1/8 & 0 & -1/8 & -1/8 & 0 & 1/4 & 1/4 & 1/4 \\
0 & 0 & 0 & 0 & 0 & 3/4 & -1/4 & -1/4 & -1/4 & 0 & 0 & 0 & 1/4 & -1/8 & -1/8 & 1/4 & -1/8 & -1/8 & 1/4 & -1/8 & -1/8 \\
0 & 0 & 0 & 0 & 0 & -1/4 & 3/4 & -1/4 & -1/4 & 1/4 & -1/8 & -1/8 & 0 & 0 & 0 & -1/8 & 1/4 & -1/8 & -1/8 & 1/4 & -1/8 \\
0 & 0 & 0 & 0 & 0 & -1/4 & -1/4 & 3/4 & -1/4 & -1/8 & 1/4 & -1/8 & -1/8 & 1/4 & -1/8 & 0 & 0 & 0 & -1/8 & -1/8 & 1/4 \\
0 & 0 & 0 & 0 & 0 & -1/4 & -1/4 & -1/4 & 3/4 & -1/8 & -1/8 & 1/4 & -1/8 & -1/8 & 1/4 & -1/8 & -1/8 & 1/4 & 0 & 0 & 0 \\
0 & 0 & 0 & 0 & 0 & 0 & 0 & 0 & 0 & 5/12 & -5/24 & -5/24 & -1/12 & 1/24 & 1/24 & 1/24 & -5/24 & 1/6 & 1/24 & -5/24 & 1/6 \\
0 & 0 & 0 & 0 & 0 & 0 & 0 & 0 & 0 & -5/24 & 5/12 & -5/24 & 1/24 & -5/24 & 1/6 & -1/12 & 1/24 & 1/24 & 1/24 & 1/6 & -5/24 \\
0 & 0 & 0 & 0 & 0 & 0 & 0 & 0 & 0 & -5/24 & -5/24 & 5/12 & 1/24 & 1/6 & -5/24 & 1/24 & 1/6 & -5/24 & -1/12 & 1/24 & 1/24 \\
0 & 0 & 0 & 0 & 0 & 0 & 0 & 0 & 0 & -1/12 & 1/24 & 1/24 & 5/12 & -5/24 & -5/24 & -5/24 & 1/24 & 1/6 & -5/24 & 1/24 & 1/6 \\
0 & 0 & 0 & 0 & 0 & 0 & 0 & 0 & 0 & 1/24 & 5/24 & 1/6 & -5/24 & 5/12 & -5/24 & 1/24 & -1/12 & 1/24 & 1/6 & 1/24 & -5/24 \\
0 & 0 & 0 & 0 & 0 & 0 & 0 & 0 & 0 & 1/24 & 1/6 & -5/24 & -5/24 & -5/24 & 5/12 & 1/6 & 1/24 & -5/24 & 1/24 & -1/12 & 1/24 \\
0 & 0 & 0 & 0 & 0 & 0 & 0 & 0 & 0 & 1/24 & -1/12 & 1/24 & -5/24 & 1/24 & 1/6 & 5/12 & -5/24 & -5/24 & -5/24 & 1/6 & 1/24 \\
0 & 0 & 0 & 0 & 0 & 0 & 0 & 0 & 0 & -5/24 & 1/24 & 1/6 & 1/24 & -1/12 & 1/24 & -5/24 & 5/12 & -5/24 & 1/6 & -5/24 & 1/24 \\
0 & 0 & 0 & 0 & 0 & 0 & 0 & 0 & 0 & 1/6 & 1/24 & -5/24 & 1/6 & 1/24 & -5/24 & -5/24 & -5/24 & 5/12 & 1/24 & 1/24 & -1/12 \\
0 & 0 & 0 & 0 & 0 & 0 & 0 & 0 & 0 & 1/24 & 1/24 & -5/24 & -5/24 & 1/6 & 1/24 & -5/24 & 1/6 & 1/24 & 5/12 & -5/24 & -5/24 \\
0 & 0 & 0 & 0 & 0 & 0 & 0 & 0 & 0 & -5/24 & 1/6 & 1/24 & 1/24 & 1/24 & -1/12 & 1/6 & -5/24 & 1/24 & -5/24 & 5/12 & -5/24 \\
0 & 0 & 0 & 0 & 0 & 0 & 0 & 0 & 0 & 1/6 & -5/24 & 1/24 & 1/6 & -5/24 & 1/24 & 1/24 & 1/24 & -1/12 & -5/24 & -5/24 & 5/12
\end{bmatrix}
\begin{bmatrix}
Fam \\ Act_M \\ Act_F \\ Act_{C1} \\ Act_{C2} \\ Par_M \\ Par_F \\ Par_{C1} \\ Par_{C2} \\ Rel_{MF} \\ Rel_{MC1} \\ Rel_{MC2} \\ Rel_{FM} \\ Rel_{FC1} \\ Rel_{FC2} \\ Rel_{C1M} \\ Rel_{C1F} \\ Rel_{C1C2} \\ Rel_{C2M} \\ Rel_{C2F} \\ Rel_{C2C1}
\end{bmatrix}
$$

We can now use this product to work out the concrete link between the mean of the ANOVA score for the mother's actor effect $\overline{Act_M}$ and the factor mean of the mother's actor effect Act as follows

$$\overline{Act_M} = \frac{3}{4}Act_M - \frac{1}{4}Act_F - \frac{1}{4}Act_{C1} - \frac{1}{4}Act_{C2} + \frac{1}{4}(Rel_{MF} + Rel_{MC1} + Rel_{MC2}) - \frac{1}{8}(Rel_{FC1} + Rel_{FC2}) - \frac{1}{8}(Rel_{C1F} + Rel_{C1C2}) - \frac{1}{8}(Rel_{C2F} + Rel_{C2C1})$$

We know that the sum of the relationship effects for the given actor effect of the mother sum to zero, thus$(Rel_{MF} + Rel_{MC1} + Rel_{MC2})$ will be equal to zero. The relationship constraint also implies that two relationship effects given a same actor effect can be expressed as the third relationship effect given that actor. Meaning that, for example, the sum $(Rel_{FC1} + Rel_{FC2})$ can be replaced by $-Rel_{FM}$. Thus, the link can be rewritten as

$$\overline{Act_M} = \frac{3}{4}Act_M - \frac{1}{4}Act_F - \frac{1}{4}Act_{C1} - \frac{1}{4}Act_{C2} + \frac{1}{8}(Rel_{FM} + Rel_{C1M} + Rel_{C2M})$$

We also know that the sum of the relationship effects for a given partner effect sum to zero, meaning that here the sum $(Rel_{FM} + Rel_{C1M} + Rel_{C2M})$ will equal zero as well. In an equivalent manner, we can make use of the constraint on the actor effects to simplify this equation even more. Given that the sum of the actor effects is zero, the sum of the actor effect of the father, oldest child and youngest child can be expressed in terms of the actor effect of the mother. This entails that we can easily show that mean of the ANOVA score and the factor mean coincide as follows

$$\overline{Act_M} = \frac{3}{4}Act_M - \frac{1}{4}(Act_F + Act_{C1} + Act_{C2})$$

$$= \frac{3}{4}Act_M + \frac{1}{4}Act_M$$

$$= Act_M$$

The link between the mean of the ANOVA scores of the other actor effects and the partner effects and their accompanying factor means can be worked out in a similar fashion.

Next, we can do something similar for the link between the mean of the ANOVA score for the M-C2 relationship effect $\overline{Rel_{MC2}}$ and the factor mean of the M-C2 relationship effect Rel_{MC2}. Here, we make use of the constraints on the relationship effects again.

$$\overline{Rel_{MC2}} = -\frac{5}{24}(Rel_{MF} + Rel_{MC1}) + \frac{5}{12}Rel_{MC2} + \frac{1}{24}Rel_{FM} + \frac{1}{6}Rel_{FC1} - \frac{5}{24}Rel_{FC2}$$
$$+ \frac{1}{24}Rel_{C1M} + \frac{1}{6}Rel_{C1F} - \frac{5}{24}Rel_{C1C2} - \frac{1}{12}Rel_{C2M} + \frac{1}{24}Rel_{C2F} + \frac{1}{24}Rel_{C2C1}$$
$$= -\frac{5}{24}Rel_{MC2} + \frac{5}{12}Rel_{MC2} + \frac{1}{24}(Rel_{FM} + Rel_{C1M}) - \frac{5}{24}(Rel_{FC2} + Rel_{C1C2})$$
$$+ \frac{1}{24}(Rel_{C2F} + Rel_{C2C1}) + \frac{1}{6}Rel_{FC1} + \frac{1}{6}Rel_{C1F} - \frac{1}{12}Rel_{C2M}$$

$$= -\frac{15}{24}Rel_{MC2} - \frac{1}{24}Rel_{C2M} + \frac{5}{24}Rel_{MC2} - \frac{1}{24}Rel_{C2M} + \frac{1}{6}Rel_{FC1} + \frac{1}{6}Rel_{C1F}$$
$$- \frac{1}{12}Rel_{C2M}$$

$$= \frac{20}{24}Rel_{MC2} - \frac{1}{6}Rel_{C2M} + \frac{1}{6}Rel_{FC1} + \frac{1}{6}Rel_{C1F}$$

$$= \frac{20}{24}Rel_{MC2} + \frac{1}{6}(Rel_{C2F} + Rel_{C2C1}) + \frac{1}{6}Rel_{FC1} + \frac{1}{6}Rel_{C1F}$$

$$= \frac{5}{6}Rel_{MC2} + \frac{1}{6}(Rel_{C2F} + Rel_{C2C1}) + \frac{1}{6}(Rel_{C2C1} + Rel_{FC1})$$

$$= \frac{5}{6}Rel_{MC2} - \frac{1}{6}Rel_{MF} - \frac{1}{6}Rel_{MC1}$$

$$= \frac{5}{6}Rel_{MC2} - \frac{1}{6}(Rel_{MF} + Rel_{MC1})$$

$$= \frac{5}{6}Rel_{MC2} + \frac{1}{6}Rel_{MC2}$$

Further, the covariance matrix of the estimated ANOVA scores $\Sigma_{\hat{f}}$ is related to the covariance matrix of the latent SRM effects Ψ as follows (Hoshino & Bentler, 2011)

$$\Sigma_{\hat{f}} = W(\Lambda\Psi\Lambda' + \Theta)W' \qquad (14)$$

where Θ refers to the residual covariance matrix. Since the residual covariance matrix of the SRM is zero, it is redundant in this equation. Thus, the covariance matrix of the estimated ANOVA scores can here be expressed as:

$$\Sigma_{\hat{f}} = W(\Lambda\Psi\Lambda')W' \qquad (15)$$

Based on this expression, we can deduce that the covariance matrix of the estimated scores will only equal Ψ if the product of the weight matrix W and the factor loading matrix Λ results in an identity matrix. However, this is generally not the case for the SRM. The covariance matrix of the estimated SRM ANOVA scores will thus give biased estimates of the true SRM variances.

Consequently, the sample's variance of the ANOVA actor effect will show a discrepancy with the estimate of the actor variance obtained using a CFA.

Hence, in the calculation of the Z scores we cannot simply replace the ANOVA standard deviations with the CFA standard deviations, since this could lead to the wrong conclusions. Thus, to obtain Z scores that are comparable to those of the original approach of Cook and Kenny (2004) we need to transform the variances obtained with the CFA. Given that the ML-estimator of the covariance matrix of the CFA is an asymptotically unbiased estimate of the true covariance Ψ, we can estimate the covariance matrix of the SRM ANOVA scores by replacing Ψ in expression (16) with its ML-estimator from the CFA. Simply put, we will transform the covariance matrix of the CFA to obtain an estimate of the variance of the ANOVA scores.
